# Isotretinoin Use in Transmasculine Patients and Its Implication on Chest Masculinization Surgery: Scoping Review of the Literature

**DOI:** 10.2196/45351

**Published:** 2023-08-10

**Authors:** Daniel Strock, Torunn E Sivesind, Robert P Dellavalle, Gerhard S Mundinger

**Affiliations:** 1 School of Medicine Eastern Virginia Medical School Norfolk, VA United States; 2 Department of Dermatology University of Colorado Anschutz Medical Campus Aurora, CO United States; 3 Department of Epidemiology Colorado School of Public Health University of Colorado Anschutz Medical Campus Aurora, CO United States; 4 Dermatology Service Rocky Mountain Regional Veterans Affairs Medical Center Aurora, CO United States; 5 Crane Center for Transgender Surgery Austin, TX United States

**Keywords:** acne, isotretinoin, transsexual, scoping, post operative, surgical, pharmacology, pharmaceutic, retinoid, testosterone therapy, sex reassignment, gender affirmation surgery, masculinization surgery, gender reassignment, derm, dermatology, dermis, oily skin, hormone therapy, perioperative, operation, scar, keloid, surgery, surgeries, trans, transmen, transgender, transex, top surgery, chest masculinization, accutane, roaccutane

## Abstract

**Background:**

Acne often worsens in transmasculine patients who are on prolonged testosterone therapy. Isotretinoin is an oral retinoid used in the treatment of severe or refractory cases of acne, but it has the potential to cause delayed wound healing. Transmasculine patients may potentially be prescribed treatment for acne with isotretinoin while also planning to undergo chest masculinization surgery.

**Objective:**

This scoping review aims to determine whether isotretinoin has a negative impact on postoperative healing in transmasculine patients undergoing chest masculinization surgery.

**Methods:**

A scoping review was performed using the PubMed and Ovid databases. A total of 16 publications were selected for inclusion.

**Results:**

Acne tends to peak in transmasculine patients 6 months after initiation of testosterone treatment. Severe cases can be treated with isotretinoin; however, acne may recur once treatment is discontinued, given ongoing hormone therapy. There is little to no evidence in the medical literature regarding perioperative use of isotretinoin specifically among transmasculine patients undergoing chest masculinization surgery. In general, however, recent studies have found no evidence of increased hypertrophic scars or keloids in patients taking isotretinoin.

**Conclusions:**

Further studies are required to strengthen the current evidence that suggests that isotretinoin does not need to be discontinued before or after incisional or excisional surgeries, including chest masculinization surgery in transmasculine patients.

## Introduction

Acne is an inflammatory condition of the skin commonly treated by dermatologists. While the pathogenesis of acne is multifactorial, recent research has emphasized the importance of hormones in the function of the pilosebaceous unit [1]. In sebaceous glands, testosterone is converted to dihydrotestosterone and binds to cytoplasmic androgen receptors, promoting epidermal growth and sebocyte proliferation. This increased sebum production plays a principal role in the development of acne. This is of particular relevance to female-to-male transgender patients (herein referred to as “transmasculine patients”) who are often prescribed masculinizing doses of exogenous testosterone as part of gender-affirming hormone therapy. Isotretinoin may be prescribed for more severe or refractory cases of acne, but it has the potential to delay wound healing due to its effect on the stem cells responsible for repopulating the epidermis. Studies in animal models have demonstrated isotretinoin’s effect on the pilosebaceous unit, whereby the sebaceous glands were greatly reduced in volume [2]. Given that transmasculine patients may pursue bilateral mastectomy and chest masculinization surgery (often referred to as “top surgery”) as a means of gender affirmation, it is vital for dermatologists to be aware of how isotretinoin use may impact chest masculinization surgery in this population. A scoping review [3] of relevant literature available via a web-based database search was performed in order to assist clinicians in making informed, evidence-based decisions with their patients and to identify any existing knowledge gaps regarding isotretinoin treatment and chest masculinization surgery.

## Methods

A search of the available biomedical literature was performed using the PubMed database on September 1, 2022. An initial query using the search string, “(transmen OR “trans men” OR trans-men OR transgender* OR transex* OR transmasc*) AND (“top surgery” OR “Chest masculinization”) AND (isotretinoin OR accutane OR roaccutane),” yielded zero results. A more focused search used the string, “(transmen OR “trans men” OR trans-men OR transgender* OR transex* OR transmasc*) AND (isotretinoin OR accutane OR roaccutane),” yielded 19 results, all of which were published between 2015 and 2022. Another search using the string, “(surg*) AND (heal* OR scar*) AND (isotretinoin OR accutane OR roaccutane),” limited to publications within 10 years, yielded 109 results, for a combined total of 128 articles selected for title and abstract screening. This was performed by 2 independent reviewers (DS and TS), with 40 articles selected for subsequent full-text review. After full-text review, a total of 11 studies were selected for inclusion. Surveying the references of these 11 articles revealed an additional 5 relevant studies, which were also chosen for inclusion.

An additional search was later conducted using the Ovid database on June 18, 2023. The search terms “(transmen OR “trans men” OR trans-men OR transgender* OR transex* OR transmasc*) AND (“top surgery” OR “Chest masculinization”) AND (isotretinoin OR accutane OR roaccutane)” yielded zero results. A search using the key terms “(transmen OR “trans men” OR trans-men OR transgender* OR transex* OR transmasc*) AND (isotretinoin OR accutane OR roaccutane)” yielded 17 results, though no articles were new in respect to the previously conducted PubMed search. The key terms “(surg*) AND (heal* OR scar*) AND (isotretinoin OR accutane OR roaccutane),” limited to publications within 10 years, yielded 33 results, all of which were similarly included in the previous PubMed search.

## Results

### Acne and Isotretinoin Use in Transmasculine Patients

Masculinizing hormone therapy used in transmasculine patients has the potential to worsen acne, mediated largely by testosterone’s effect on the pilosebaceous unit [4]. In a prospective study of 20 transmasculine patients undergoing exogenous testosterone therapy, rates of facial acne increased from 35% to 82.4% following 6 months of treatment, whereas rates of chest and back acne increased from 15% to 88.2% within the same time frame [4]. Another prospective cohort study of 193 transmasculine patients demonstrated that self-reported scores of acne severity peaked 6 months after initiating testosterone therapy, and were still significantly higher than those at baseline after 12 months of therapy [5]. A retrospective review of medical records of 55 transmasculine patients also demonstrated a significant association between acne and a serum testosterone level greater than 630 ng/dL [6].

No evidence-based guidelines currently exist for treating acne in the context of testosterone therapy [7]. Though acne caused by exogenous testosterone is often mild to moderate in severity [8], more persistent or extensive cases may necessitate treatment with isotretinoin. While isotretinoin can be an appropriate treatment for acne in the setting of prolonged testosterone therapy, there are many special considerations when prescribing isotretinoin for transmasculine patients [9]. Given the teratogenicity of isotretinoin, clinicians should be prepared to engage their patients in thorough but sensitive conversations regarding their risk of pregnancy. Additionally, though the relationship between isotretinoin use and idiosyncratic depression is largely controversial, it should be noted that depressive symptoms, suicidal ideation, and self-harm are more common in transgender individuals. These comorbidities are posited to be largely the result of social stresses including bullying, harassment, social stigma, and decreased support from family members [10]. It also unclear whether long-term remission can be achieved with one “cycle” of isotretinoin in the context of ongoing testosterone therapy. No large-scale randomized studies have examined isotretinoin use in this specific population, but several case studies have demonstrated the efficacy of isotretinoin therapy in the treatment of testosterone-dependent acne; these results are presented in Table 1 [11,12].

All 4 patients presented in these case studies had a positive response to isotretinoin therapy. Patients 1 and 2 achieved resolution of their acne, but ultimately presented with recurrence necessitating ongoing isotretinoin therapy. Patient 3 achieved remission and decided to discontinue treatment at 4 months. Patient 4 demonstrated partial clearance of his acne, but associated therapy with a depressive episode and discontinued at 3.5 months of treatment.

**Table 1:** Case series of transgender males treated with isotretinoin [11,12].

Patient number	Patient age (years)	Testosterone dose	Length of testosterone treatment before starting isotretinoin	Isotretinoin dose
1	20-29^a^	Testosterone undecanoate, 1000 mg every 3 months	6 months	30 mg/day for 9 months, discontinue for 3 months, ongoing at 20 mg/day
2	20-29^a^	Testosterone undecanoate, 1000 mg every 3 months	6 months	20 mg/d for 8 months, discontinue for 6 months, ongoing at 20 mg 3 times per week
3	17	Testosterone (formulation unspecified), 250 mg every 21 days	6 months	20 mg/day for 3.5 months
4	17	Testosterone (formulation unspecified), 250 mg every 21 days	5 months	20 mg/day for 4 months

^a^The patient in the original case report was referred to as being “in his 20s.”

### Isotretinoin and Surgical Healing

It has traditionally been taught that surgical procedures should not be performed while a patient is taking oral isotretinoin, given concerns that retinoids may alter connective tissue healing or lead to keloids or hypertrophic scarring. These recommendations are based largely on 3 case series published in the 1980s, which reported the formation of keloids in 9 patients on isotretinoin therapy, who subsequently underwent dermabrasion or argon laser phototherapy [13,14]. However, recent literature on the subject contradicts these earlier reports, as noted by the American Society for Dermatologic Surgery (ASDS).

The ASDS guidelines (published in 2017) for following comprehensive literature review and task force consensus state, “The data for incisional and excisional cutaneous surgery on isotretinoin is insufficient to make any recommendations. In particular cases, incisional or excisional surgery may be medically necessary in patients receiving isotretinoin” [15]. Quality of Evidence was assigned a “D” score by task force members, using the GRADE (Grading of Recommendations, Assessment, Development, and Evaluation) system, and representing “very low” confidence in their final recommendation [15]. Another systematic review with consensus recommendations, also published in 2017, determined that there is “insufficient evidence to delay cutaneous surgery for patients currently taking or having recently completed isotretinoin therapy” [16].

More recently, a randomized controlled trial of 303 patients, published in 2020, examined the effects of perioperative isotretinoin therapy on rhinoplasty outcomes [17]. This study suggests that isotretinoin caused no significant disturbances in postoperative healing, with no hypertrophic scarring or cartilaginous deformities. A case study published in 2022 demonstrated normal scar tissue healing in 2 cisgender females who underwent bilateral reduction mammoplasty while on isotretinoin therapy [18]. Neither patient had a family history of keloids or hypertrophic scarring. Our review did not reveal any studies that specifically examined postoperative healing in transmasculine patients on isotretinoin therapy undergoing chest masculinization surgery.

## Discussion

Acne is common in transmasculine patients undergoing masculinizing hormone therapy, and some individuals with testosterone-induced acne may benefit from isotretinoin therapy. In a survey of transgender individuals, those who self-identified as men were more likely to prioritize treatment of the chest over that of their face or genitals [19]. In a cross-sectional analysis of 90 transgender men, exogenous testosterone demonstrated a significant effect on the presence of chest acne, which was present in 52% of transmasculine patients on testosterone therapy, compared to 9% of those who were not on testosterone therapy. Given that exogenous testosterone therapy has a prominent effect on the chest [20], it is vital for clinicians to recognize the impact that acne treatment has on patients’ body image–related quality of life.

When initiating isotretinoin therapy in transgender male patients, it should be standard practice for dermatologists to discuss plans for any upcoming surgery, including chest masculinization surgery, with their patients. While current literature does not suggest any effect of isotretinoin on postsurgical wound healing, risks and benefits of perioperative treatment should be discussed with patients, and a treatment plan should be formulated on the basis of their individual goals of care and severity of acne. Given the lack of specific evidence in this patient population, surgery would ideally be performed before initiation of isotretinoin therapy in order to minimize any interactions. However, in cases of severe acne with a risk of scarring, it is not recommended to delay treatment with isotretinoin or to discontinue isotretinoin before chest masculinization surgery.

Further research is needed on this particular subject matter and patient population in order to better to characterize the exact relationship between isotretinoin use and chest masculinization surgery, so that dermatologists may better coordinate care for their transgender patients.

## Abbreviations

ASDS: American Society for Dermatologic Surgery
GRADE: Grading of Recommendations, Assessment, Development, and Evaluation
